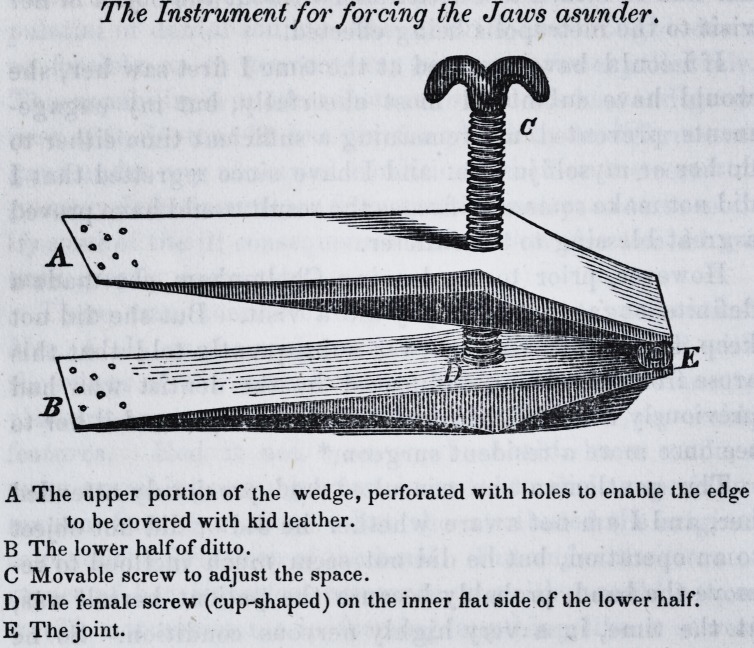# Anchylosis of the Jaws from a Semi-Cartilaginous Band Connecting the Upper and Lower Jaws

**Published:** 1860-04

**Authors:** J. L. Levison

**Affiliations:** London.


### ARTICLE VII.

*Anchylosis of the Jaws from a Semi-Cartilaginous Band Connecting the Upper and Lower Jaws.* By DR. J. L. LEVISON, of London.

SOME years since, during a hurried visit to Cheltenham, I was asked by a relative of mine to see a friend of hers, "who had some extraordinary growth in her mouth, which effectually prevented her from opening her mouth."

On the following day the lady was introduced to me, when I made a careful examination of her mouth, the particulars of which I shall now detail:

Mrs. A. J., a married woman, about forty, (without any family,) and of a nervo-bilious temperament; in her appearance there was nothing to indicate any abnormal tendency, and her particular annoyance was only manifested when she attempted to answer some questions put to her. I found on the left side of her mouth a strong semi-cartilaginous band, which seemed to originate in the upper jaw. The situation of this band being about an inch from the corner of the mouth and the insertion of the buccinator muscle near the dens molars, and probably also intimately connected with the masseter; as the latter arises by strong tendinous and fleshy fibres from the superior maxillary bone, where it joins the os malæ, the whole length of the inner edge of the zygomata. And the probable connection of the band with the latter muscle may be fairly presumed, as the outer part of the masseter stands backwards, and the inner part forwards, and in some measure decussating each other. In its descent it covers the coronoid process under the temporal muscles to be inserted in the angle of the lower jaw, and from upwards to the outside of the coronoid process.

The probable origin of this pseudo-growth may be further confirmed by the fact, that the action of the buccinator is to draw the mouth backwards and outwards, and to contract the cavity by pressing the cheeks inwards; whilst the action of the masseters is to raise the lower jaw, and pull it backwards and forwards, according to the direction of the fibres of these muscles.

We may now speak of this freak of nature and point out the many annoying consequences it induced.

The poor creature's teeth approximated so closely that she could neither cut, tear, or grind her food, and had to live on slops; besides this her pronunciation of words was seriously defective.

The first defect was attempted to be obviated by the extraction of an upper and a lower molar, on the opposite side of the mouth at which the band was situated, for the purpose of expediting, by means of a curved tube, her capacity of receiving mucilaginous and other nutritious fluids.

But little advantage actually resulted, and for this reason: that it is essential, in order to produce a vacuum, for the purpose of deglutition, the lips should be closed, and the soft palate pressed up behind the nasal fossæ, and then both fluids and solids are propelled onwards and received into the pharynx, and then the final act of swallowing takes place by the action of the muscles of the latter organ.

The cause of her defective speaking is also worthy of a passing notice. She spoke precisely like a person who has a fissure in the palatial bones, as in congenital hare-lip; in which case the bones are more or less separated, and the pronunciation is more or less implicated.

In the normal state of the vocal organs, when the air inspired by the lungs is used to form the vocal sounds—during the act of expiration, the different organs of the buccal cavity modify and individualize each kind—as the teeth, lips, and the palate, producing dentals, labials, and palatials, and these elementary sounds are combined, their compounds are the dento-labial, dento-palatial, and so forth.

But in the attempted speech of Mrs. A. J., the sounds passed from the back of the mouth through the nostrils, and these sounds were, consequently, as indistinct as if she had had a congenital defect of the ossa-palati, to a considerable degree.

What tended to make her speech appear to be defective from this cause simply, arose from the fact, that in cases of fissures of the palate there is observed a curious attempt to compensate for the condition of the sounding board, as the roof of the mouth has been called; this compensation enables an individual so circumstanced to obtain a perfect volition over both nostrils, so that in the utterance of the palatial or dental sounds—the nostrils are brought down so forcibly as to prevent any escape of the expelled air. This capability is another instance of the wisdom and goodness of God—that he has given a plasticity to different organs under any abnormal condition, whether the result of primary defectiveness, or from some casualty, so as to modify some of the ill consequences which otherwise would have been greatly aggravated.

There was, therefore, a great difficulty to understand Mrs. A. J., as she spoke with a constant snuffle, and with the wings of the nose so compressed, as to impart an unpleasant appearance to one who had, otherwise, agreeable features. Had it not been so, I should have made a more searching examination in order to elicit some information from her as to what she attributed the original cause of the formation of the band. But under the circumstance of her imperfect utterance, she either could not, or would not assist me in forming anything like a correct *diagnosis*.

Nevertheless, being satisfied that the band might be safely separated, I proposed that she should visit me at Brighton, (where I then resided,) when I proposed to operate on her and remove the band without much pain or any danger. And that to ensure both, I would place her under the medical treatment of my friend, Dr. Peckford. And further explained to her that my object would be, not only to remove the band, but to prevent its future growth.

She said, "I have the greatest confidence in your judgment, for your opinion exactly coincides in every particular with the opinion given to me by the late Mr. Liston, whom I consulted about a fortnight before his death. He told me at the time, that being much engaged, he would prefer seeing me on a future day, as a little delay would not render the operation more difficult.

But it appeared that before the period he had appointed to see her again, that gifted surgeon had paid the debt of nature, and his death had caused her great sorrow; for she had to return to Cheltenham without the object of her visit to the metropolis being effected.

If I could have operated at the time I first saw her, she would have submitted most cheerfully, but my engagements prevented my remaining a sufficient time either to do her or myself justice: and I have since regretted that I did not make some sacrifice, as the result would have proved a great blessing to the sufferer.

However, prior to my leaving Cheltenham, she made a definite engagement to pay me a visit. But she did not keep her word. And I was subsequently told that this arose from her being influenced by the dentist who had previously attended her, and that he had persuaded her to see once more a resident surgeon.*

The gentleman she consulted had previously attended her, and I am not aware whether he did or did not object to an operation, but he did not seem much inclined to remove the band, probably because the patient herself was, at the time, in a very highly nervous condition. So he substituted a mechanical contrivance, but for what object I cannot opine. As any force which could be applied without immediate danger would not have prevented the anchylosis, so long as the resisting cartilaginous band remained.

This instrument I subsequently inspected. It was of a

* This gentleman's name was S., and he has since then departed from this world, rather prematurely, for he was a young man.

wedge form, being made of two steel portions, graduated, so as to be flat and thinnest at the edges. The flat ends were perforated with holes, for the purpose of being covered with white kid leather. A screw was placed through the upper half of the wedge, to terminate into a cup-like female screw, affixed to the inner part of the lower half of it; and so contrived that the spaces between the edges of the wedge could be enlarged at the will of the patient. The edges of which were inserted or rather forced between front teeth.

*The Instrument for forcing the Jaws asunder.*

A The upper portion of the wedge, perforated with holes to enable the edge to be covered with kid leather.
B The lower half of ditto.
C Movable screw to adjust the space.
D The female screw (cup-shaped) on the inner flat side of the lower half.
E The joint.

Mrs. A. J. persevered for two months in using this instrument, until she experienced great agony, and not finding the slightest improvement, she abandoned its use altogether. And when the nature of the strong resisting band is taken into consideration, and that it utterly prevented the action of the *temporales* and *masseters*, no other result could have beon anticipated.

Soon after this experiment, I received from my correspondent a reply to certain queries of mine, and among others the following:

"Mrs. A. J. has been very ill this winter from an enlarged lung on one side, and under the slightest cold she is incapacitated to speak, even in her usually imperfect manner. She is now suffering from bronchitis, and looks very ill and much emaciated."

It seems to me not improbable that the wedge was intended to gradually separate the jaws, being forced between the teeth ; and it may be presumed that the pain she complained of whilst wearing the instrument, had induced a *chronic* inflammation of the integuments and muscles of the buccal cavity, causing both the latter to become thickened, so far as may be judged of from her subsequent symptoms, and the results.

I saw her again, about two years after the wedge experiment, and my own first examination, (now about six years since,) and found the band, instead of being separated from the inner portion of the cheek, so to admit the finger passing freely between it and the band, the latter had become so perfectly united with the cheek, that it would now be impossible to do any good by any attempt to separate it ; and it could only be removed by dissecting it away from the cheek to which it is incorporated, and in so doing, as it is merely covered with the integument, any operation might tend to induce another very unpleasant deformity.

The last spring, having had occasion to visit Cheltenham again, on a melancholy bereavement of one of my family, I took the opportunity of once more examining the patient, and now feel assured that any operation would be attended with some danger.

The jaws are perfectly immovable, and the *anchylosis* is complete. And although she lives altogether on suction, yet she looked better in health than I had anticipated. There is, however, every probability that she may ultimately die from actual inanition.

As my object in giving publicity to this *unique* case is for the purpose of obtaining some information on its probable origin, I shall conclude, therefore, this brief paper with a summary of an article on anchylosis of the alveolar arch, in Oppenheim's Zeitschrift Band, 44, page 375, as given in the xviith number of the Medico-Chirurgical Review, for January, 1852.

The writer says, "anchylosis of the lower jaw may occur in three localities. 1st. The head of the condyle may become fixed in the glenoid cavity. This form is the most frequent, examples of which are recorded by Sandifort, Blandon, Cruvelhier, Howship, Holcher, Hyrtle, and others.

"2d. The coronoid process may become attached to the zygomatic arch, of this two cases are recorded.

"3d. The alveolar process may become conjoined.* Of this form there are examples besides the one recorded by Dr. Werner, which is now cited. "S. R., at twenty-three; when three years old he underwent severe salivation, after which his jaw remained in a fixed state. Notwithstanding the absence of masticatory powers, he was well nourished. The jaw was quite immovable, firm pressure or traction exerting no effect on the condition of the teeth. The *incisores* and *molares* were indeed for the most part wanting, the roots of which did exist, projecting beyond the alveoli of the diminutive jaw bone. The jaws were so far separated, that with some trouble, a little finger could be introduced in front, but from the anterior on each side backwards, bony arches connected the upper and lower jaws. The buccal mucous membrane was attached to the gums at the edges of these arches, but the temporal and masseter muscles remained free.

"Speech much resembled that which takes place with the

* I have seen a preternatural growth of gums, which gums have completely hidden the teeth, falling down over them like a curtain. In this disease of the *alveoli*, they seem to suffer also from an abnormal growth.

mouth closed; and food which did not require mastication was introduced between the defective teeth.

"To remedy this state of things, the gums were separated by an incision from the cheeks to the lips, and a broad portion of the connecting arch on either side removed by a small saw. The jaws could not be expanded by the aid of a mouth speculum, to the extent of half an inch, some painful stretching of the muscles being induced. The patient was, however, enabled to voluntarily close the mouth again—proving that more than twenty years' inactivity had not destroyed the functions of the joints and muscles. After several weeks perseverance in gradual dilation, a still wider expansion was obtained, enabling the patient to chew food that was not too hard, which indeed the loose state of his teeth prevented from biting."

[Vide Medical and Chirurgical Review.]

Mrs. A. J. was, in all probability, more than thirty before the band was formed, and now, after the lapse of so many years, there is every probability that both the muscles and joints in her case are implicated.

As, therefore, *anchyloses* of the jaws are rare, even when dependent on morbid growth of the alveoli, or the union of the coronoid process in the glenoid cavity, little can be known of their special pathology, and in the case of Mrs. A. J., (now first reported,) it being altogether an isolated one, the difficulty is still greater. 1st. As to origin of the band, and what predisposed its peculiar formation. But we have *data* to indicate the cause which aggravated the inconvenience and destroyed all chances of cure; namely, the pressure and force of the machine, and the consequential adhesion of the band to the cheek itself, from which it had been previously separated. Those who are acquainted with the organs of the buccal cavity, and particularly those who supply artificial teeth, know that often, in narrow jaws, (commonly spoken of as rabbit mouthed,) that elastic springs will often excoriate the inner surfaces of the cheeks, and that an inflammatory condition thus set up will affect the *temporales*, &c., by sympathy, causing much pain to the patient. Hence the mechanical force induced by the wedge instrument, not only caused inflammation of the muscles and the integuments of the cheek, and by thus thickening them, point out with certainty why now the band and cheek are so intimately united. And that though when not so connected, there would not have been any danger to operate, that now it would be dangerous to attempt any.

## Figures and Tables

**Figure f1:**